# The prevalence of unintended pregnancy and its association with HIV status among pregnant women in South Africa, a national antenatal survey, 2019

**DOI:** 10.1038/s41598-021-03096-z

**Published:** 2021-12-09

**Authors:** Selamawit Woldesenbet, Tendesayi Kufa, Carl Lombard, Samuel Manda, Diane Morof, Mireille Cheyip, Kassahun Ayalew, Adrian Puren

**Affiliations:** 1 Center for HIV and STI, National Institute for Communicable Diseases, 1 Modderfontein road, Sandringham, Johannesburg, 2192 South Africa; 2 School of Public Health, University of the Witwatersrand, Johannesburg, South Africa; 3 Biostatistics Unit, South African Medical Research Council, Cape Town, South Africa; 4 Biostatistics Unit, South African Medical Research Council, Pretoria, South Africa; 5 Department of Statistics, University of Pretoria, Pretoria, South Africa; 6 Associate Director for Science, Division of Global HIV/AIDS and Tuberculosis, U.S. Centers for Disease Control and Prevention, Pretoria, South Africa; 7 United States Public Health Service Commissioned Corps, Rockville, MD USA; 8 Division of Global HIV/AIDS and Tuberculosis, U.S. Centers for Disease Control and Prevention, Pretoria, South Africa; 9 Division of Virology, School of Pathology, University of the Witwatersrand, Johannesburg, South Africa

**Keywords:** Infectious diseases, Paediatrics, Epidemiology

## Abstract

To describe the prevalence of unintended pregnancy and its association with HIV status among pregnant women in South Africa. A cross-sectional survey was conducted between October and mid-November 2019 among pregnant women aged 15–49 years in 1589 selected public antenatal care facilities. Pregnancy intention was assessed using two questions from the London Measure of Unplanned Pregnancy. Survey logistic regression examined factors associated with unintended pregnancy. Among 34,946 participants, 51.6% had an unintended pregnancy. On multivariable analysis, the odds of unintended pregnancy was higher among women who knew their HIV-positive status before pregnancy but initiated treatment after the first antenatal visit (adjusted odds ratio [aOR], 1.5 [95% confidence interval (CI):1.2–1.8]), women who initiated treatment before pregnancy (aOR, 1.3 [95% CI:1.2–1.3]), and women with a new HIV diagnosis during pregnancy (aOR, 1.2 [95% CI:1.1–1.3]) compared to HIV-negative women. Women who were single, in a non-cohabiting or a cohabiting relationship, and young women (15–24 years) had significantly higher risk of unintended pregnancy compared to married women and women aged 30–49 years, respectively. A comprehensive approach, including regular assessment of HIV clients’ pregnancy intention, and adolescent and youth-friendly reproductive health services could help prevent unintended pregnancy.

## Introduction

South Africa has the largest HIV epidemic in the world with 7.8 million people living with HIV (PLHIV) in 2020^1^. According to the Joint United Nations Programme on HIV/AIDS (UNAIDS) estimate, in 2020, most (92%) PLHIV in South Africa knew their HIV status, however the percentage of PLHIV receiving antiretroviral therapy (ART) in South Africa remained lower than the regional average for Eastern and Southern African countries (72% vs 77% respectively)^1^. South Africa has made huge progress in improving the coverage of ART among pregnant women. In 2020, > 95% of HIV positive pregnant women in South Africa received ART. In the same year, mother-to-child HIV transmission rate in South Africa was 2% at 6 weeks and 4% at 18 months post child birth, a 75% drop from the rate in 2010 (16%)^2,3^.

Preventing unintended pregnancies among HIV-positive women is a key strategy in the elimination of mother-to-child HIV transmission, and helps reduce a range of other adverse maternal and child health outcomes. Unintended pregnancy is unwanted at the time of conception^4^. Globally, in average 121 million pregnancies each year between 2015 and 2019 were unintended^5^. In the same period, unintended pregnancies were higher in Sub-Saharan African (SSA) countries compared to the global average (91 vs 64 unintended pregnancies per 1000 women aged 15–49 years per annum respectively)^5^. Women with unintended pregnancy may not receive preconception care and are more likely to delay initiation of antenatal care (ANC)^6,7^. Late initiation of ANC may delay diagnosis and treatment of maternal HIV and exposes infants to perinatal HIV transmission. In addition, the literature shows unintended pregnancy is associated with reduced adherence to ART which has negative consequences on both maternal and child health outcomes^8^. Most studies in sub-Saharan African countries that have assessed unintended pregnancy do not provide prevalence estimates for both HIV-positive and HIV-negative women, and the studies that provide have limited generalizability at the country level^9–14^. The findings of these studies are inconsistent, with some studies showing no difference in the rate of unintended pregnancy by HIV status, while other studies show that HIV-positive women have higher rates of unintended pregnancy than HIV-negative women; however, most of these studies were conducted before the *Test and Treat* era, when only women with < 350 cells/mm^3^ CD4 count received ART^9–12^. Expanded coverage of ART in the *Test and Treat* era could improve pregnancy planning because HIV-positive women visit health facilities more frequently than HIV-negative women and could have better access to contraceptive services than HIV-negative women.

Unintended pregnancy has other health, social, and economic consequences^15^. Late initiation of ANC delays diagnosis of other underlying maternal health conditions (such as hypertension and diabetes) that could lead to adverse maternal and child health outcomes^15–17^. For individuals and families, unintended pregnancies could have devastating consequences, including household financial stress leading to violence within the family, poor nutrition during pregnancy, poor mental health, unsafe abortion, and poor quality of life for older siblings/children^18–20^. Unintended pregnancy among school-aged girls can result in school dropouts, depression, and low educational achievement^21^.

Lack of education, lack of employment opportunities, cultural and religious beliefs, social norms (e.g., autonomy of men in decision making), and health service–related factors (e.g., quality of care and inconsistent availability of contraception options) contribute to a high prevalence of unintended pregnancy^22–24^. Unintended pregnancy affects all reproductive age groups and populations; however, young women and unmarried women have a higher rate of unintended pregnancies than older, married women^22^. High–risk behaviors (e.g., unsafe sex) also contribute to unintended pregnancy^25^.

We assessed the prevalence of unintended pregnancy and the association between HIV status and unintended pregnancy among pregnant women in South Africa.

## Methods

### Design and population

We analyzed data from the 2019 South African National Antenatal HIV Sentinel Survey, a cross-sectional survey conducted every two years in South Africa to monitor trends in HIV prevalence among pregnant women aged 15–49 years attending ANC services in public health facilities^26^. The 2019 survey aimed to enroll 36,015 pregnant women from 1589 public health facilities selected from all districts of South Africa. This sample size was calculated for the primary objective of estimating HIV prevalence. For our secondary analysis, with the sample size calculated for the primary objective, it was possible to estimate the prevalence of unintended pregnancy at the provincial and national level and by HIV status with 1%–3% precision. This estimate was based on an assumption that the prevalence of unintended pregnancy would be 54% nationally and 50% among HIV-positive women based on existing data, a design effect of 1.5, and using 95% confidence intervals. Sites from each district were selected using stratified cluster sampling and probability proportional to size sampling methods.

### Data collection procedures

During the survey period (October 1–November 15, 2019), consenting pregnant women aged 15–49 years attending the ANC clinic for the first time or for follow-up visits during their current pregnancy were consecutively enrolled until either the required sample size was reached or until the end of the study period. The survey data was collected by nurses providing ANC service in the sentinel sites. Paper based questionnaires were used for all data collection. The following data were collected through interview: participant’s education, race, relationship with the father of the child, gravidity, and pregnancy intention using two questions adopted from the London Measure of Unplanned Pregnancy (LMUP)^27^. The questions included were LMUP item 3 “Just before I became pregnant…” with response options “I intended to get pregnant,” “My intentions kept changing,” and “I did not intend to get pregnant” and LMUP item 5 “Before I became pregnant…” with response options “The father of the child and I had agreed that we would like me to be pregnant,” “The father of the child and I had discussed having children together, but hadn’t agreed for me to get pregnant,” and “We never discussed having children together.” . Participants who could not answer the partner question (e.g. those who didn’t know who the father of the child was) were advised to skip the partner question.

We could not include all six LMUP questions because there was insufficient space on the questionnaire, and a longer questionnaire would delay provision of services as the survey questionnaire was administered by the same nurses providing ANC. These two LMUP questions were chosen for inclusion in the survey because of the initial thought that the two questions could enable to assess women’s intention and involvement of their partners in pregnancy planning. While the use of the two LMUP questions (instead of using only the women’s intention question) has slightly improved the accuracy of the estimate for unintended and intended pregnancies, as the partner question (LMUP item 5) was answered by the women, the response could be biased towards the women’s intention. Thus, the partner involvement may not have been accurately estimated in this study.

Data were also extracted from medical records, which included participant age, gestational age at first ANC visit, HIV and syphilis test results, and timing of HIV diagnosis and ART initiation. Participants received both ANC and PMTCT services before enrollment in the survey. All participants attending their first ANC visit in the current pregnancy (excluding those who already knew their HIV-positive status before pregnancy) received HIV testing as part of routine care. The HIV test result was available almost immediately. Those testing positive were initiated on ART (by the ANC nurse) on the same day as part of routine care. Once all routine services (including ART where applicable) have been provided, participants were enrolled in the survey and interviewed, and data extracted from their medical record. Detailed descriptions of the study procedures are presented in the main survey report^26^.

### Data analysis

Data were analyzed using STATA 14 (StataCorp, College Station, TX)^28^. Analysis accounted for the survey design (clustering within facilities and stratification by district) and was weighted for sample size realization and for the Statistics South Africa 2019 midyear population size of women of reproductive age (15–49 years) at the provincial level^29^. Given that sites were sampled using the probability proportional to size sampling method and that the sampling period was fixed, this provided a self-weighted sample at the district level. A population finite correction factor was added to adjust for the > 5% of facilities sampled without replacement from a finite population of about 4000 public facilities. The STATA command svyset was used to specify the variables that identify the survey design.

Participants who have not met the study enrolment criteria (i.e. participants who were outside of the age range (15**–**49 years) for inclusion in the survey, and those who have not given written consent) and participants for whom the cluster (facility) name is not completed (which is an important variable for survey analysis) and those who have not answered the two pregnancy planning questions were excluded from the current analysis. These participants had similar demographic characteristics and HIV prevalence as participants included in the analysis.

Descriptive analyses included participants’ age, gravidity, race, educational status, HIV status, and ART status (for HIV-positive women). Median and interquartile ranges were reported for continuous variables, and frequencies and percentages were reported for categorical variables. Responses for the two LMUP questions were categorized as “unintended,” “ambivalent,” and “intended” based on the proximity of the responses to the three categories. Table 1 shows the classification of responses for the two LMUP questions [note that: a response where the woman had intention to be pregnant but her partner had not agreed (or had not been discussed) was classified as “ambivalent about pregnancy” in order to differentiate from a response where both the woman and the partner had agreed to have a baby].

**Table 1:** London measure of unplanned pregnancy (LMUP) questions and categorization of responses in the 2019 South African National Antenatal HIV Sentinel Survey.

Before I became pregnant**	Just before I (mother) became pregnant*		
	I intended to become pregnant	My intention kept changing	I did not intend to become pregnant
The father of the child and I (mother) had agreed that we would like me to be pregnant	**Both responses indicate intended (35.9%)**	*One response indicated intention undecided (1.1%)*	*One response indicated not intended (3.7%)*
The father of the child and I (mother) had discussed having children together but hadn’t agreed for me to be pregnant	*One response indicated intended (2.7%)*	*One response indicated intention undecided (1.8%)*	Both responses not intended (16.9%)
We never discussed having child(ren) together	*One response indicated intended (2.0%)*	*One response indicated intention undecided (1.2%)*	Both responses indicated not intended (34.7%)

*The percentages in bracket shows the weighted distribution of participants’ response to the two LMUP questions.

**Bold font: intended pregnancy; Italic font: ambivalent about pregnancy; regular font: unintended pregnancy.

In addition, we used the scoring method recommended in Hall et al.^30^ as an alternative method to categorize responses into the three categories of intention of pregnancy (Supplementary Table 1), but because the results from both methods were highly correlated, only results from the first method are presented here. The scoring method results are included in Supplementary Fig. 1.

The HIV/ART status of participants was categorized into the following five categories for the multivariable analysis: (1) started ART before pregnancy, (2) knew HIV-positive status before pregnancy but had not initiated ART until after the first antenatal visit, (3) newly diagnosed with HIV during antenatal visit, (4) positive but timing of diagnosis/timing of ART initiation unknown, and (5) HIV negative. For descriptive analysis, HIV and ART status were separately presented. Geographical type was categorized as: (1) urban, (2) peri-urban, and (3) rural for both descriptive and multivariable analysis.

The association between demographic characteristics and the planning status of pregnancies was compared using a chi-square test. A multivariable multinomial logistic regression model was fitted to examine association between HIV/ART status and intention of pregnancy by comparing the following three groups: unintended pregnancy, ambivalent about pregnancy, and intended pregnancy (using intended pregnancy as the reference group). An alternative multivariable model was fitted with ordered logistic regression (OLR) model—however, a global test conducted to assess the proportional odds assumption of the OLR model showed the proportional odds assumption was violated. Due to this reason, multinomial logistic regression was used instead of OLR for the multivariable analysis. Adjusted odds ratios (aOR) and 95% confidence intervals (CIs) are reported from multivariable modelling. Observations with missing data for variables included in the multivariable analysis were excluded from the multivariable model. The HIV/ART status was added in the model as a primary variable of interest and adjusted for confounding variables. Variables significant at *p*-value cut off point of 0.2 in a chi2 test and other variables known a priori to be influential on the outcome were included in the multivariable model. In the final model, variables significant at *p*-value cut off point of 0.05 and other variables that have ≥ 10% effect on the odds ratio of the primary variable of interest were kept. An interaction term between gravidity and age was included in the multivariable model because there was significant interaction between these two variables. The significance of the interaction term was tested using a Wald test. For the interaction between gravidity and age, stratum-specific aOR and 95% CIs are reported. In addition, a multinomial regression was fitted for each of the two LMUP questions separately (data on the separate models were not presented because these findings were similar to those of the main model).

### Ethical considerations

Participation in the survey was voluntary, and all participants provided written informed consent. To protect the confidentiality of participants’ information, we did not include participant identification information on the data collection form. Participants could withdraw from the study at any time without affecting their treatment. Participants were not compensated for their participation. Ethical approval was obtained from the University of the Witwatersrand Human Research Ethics Committee (Medical) and the nine provincial health research ethics committees. The study protocol was reviewed and approved in accordance with the Centers for Disease Control and Prevention (CDC) human research protection procedures.

## Results

Of the 41,598 participants enrolled in the 2019 South African Antenatal HIV Sentinel Survey, a small percentage (0.2%) of the participants were outside the age range (15–49 years) for inclusion in the survey (39) or had not given written consent (50)—these were excluded from the data as they didn’t meet the study enrolment criteria. In addition, 10.2% (4235) of participants who had missing cluster (facility) name were excluded, as the cluster name is an important variable for survey data analysis. From the remaining 37,274 participants, 6.2% (2328) of participants who have not answered the two pregnancy planning questions were excluded from the current analysis—the non-response rate was similar between the two questions—3.6% for LMUP 3 and 4.3% for LMUP 5. The final dataset for this analysis included 34,946 (84%) participants. Most participants were Black Africans (89.3%), and 86.1% of participants reported their highest education level was secondary education or below (Table 2). The median age was 26 years. Most (82.2%) participants were unmarried. Of the 29.1% of HIV-positive participants, most (64.1%) had initiated ART before the current pregnancy, 4.6% knew their HIV-positive status before pregnancy but started ART after their first ANC visit, 24.1% had received a new HIV diagnosis during the ANC visit, and timing of HIV diagnosis/ART initiation was unknown for 7.2%. More than half (55.3%) of the participants reported they had no intention to become pregnant, and 40.7% reported they had intended to become pregnant. One-fifth (21.3%) of the participants reported that they had discussed having children with the child’s father but hadn’t agreed to have a child, 38.0% had never discussed having a child with the father of the child, while 40.7% had discussed having children with the child’s father and had agreed to have a child. The simultaneous distribution of responses for the two LMUP questions is provided in Table 1. All percentages on Table 1 and 2 have been weighted for sample size realization and the mid-year population size of women of reproductive age in each province.

**Table 2:** Sociodemographic and clinical characteristics of participants who responded to the pregnancy intention questions in the 2019 antenatal HIV Sentinel Survey, South Africa.

Description	Sample distribution (N = 34,946)
	Number (%)*
Median (IQR)** age in years	26 (22–31)
**Age, years**	
15–19	4245 (12.5)
20–24	9001 (27.7)
25–29	8650 (27.0)
30–34	6357 (19.9)
35–49	4148 (12.9)
Missing	2545
**Relationship with the father of the child**	
Married	5988 (17.8)
Co-habiting	9182 (28.2)
In a relationship, living apart	18,234 (50.8)
Single	1128 (3.2)
Missing	414
**Population group**	
Black African	30,758 (89.3)
Colored^†^	3588 (9.2)
Other (White, Asian)	489 (1.5)
Missing	111
**Education**	
None or primary	4182 (12.6)
Secondary	25,954 (73.5)
Tertiary	4595 (13.9)
Missing	215
**Gravidity**	
Primigravida (1)	10,879 (31.1)
Multigravida (≥ 2)	23,585 (68.9)
Missing	482
**Geographical type**	
Urban	20,735 (59.3)
Rural	11,263 (32.2)
Peri-urban	2948 (8.5)
**HIV status (per medical record data)**	
HIV-positive	10,518 (29.1)
HIV-negative	24,403 (70.9)
Missing	25
Knowledge of HIV status and ART** status before pregnancy among HIV-positive participants (n = 10,518)	
Started ART before pregnancy	6833 (64.1)
Knew HIV-positive status before pregnancy but had not initiated ART until after the first antenatal visit	494 (4.6)
Newly diagnosed with HIV during antenatal visit	2469 (24.1)
Positive but timing of diagnosis/timing of ART initiation unknown	722 (7.2)
Median (IQR)** gestational age at booking (weeks)	15 (11–20)
**Pregnancy intention question 1 (LMUP item 3)**	
I intended to get pregnant	13,646 (40.7)
My intentions kept changing	1432 (4.0)
I did not intend to become pregnant	19,868 (55.3)
Pregnancy intention question 2 (LMUP** item 5)	
The father of the child and I had agreed that we would like me to be pregnant	13,647 (40.7)
The father of the child and I had discussed having children together, but hadn’t agreed for me to get pregnant	7611 (21.3)
We never discussed having children together	13,688 (38.0)

^†^Colored refers to a multiracial ethnic group.

*Weighted percentages. Missing data excluded from the denominator when calculating percentages.

**ART, antiretroviral therapy; IQR, interquartile range; LMUP, London Measure of Unplanned Pregnancy.

### Prevalence of unintended pregnancy

**Figure 1:**
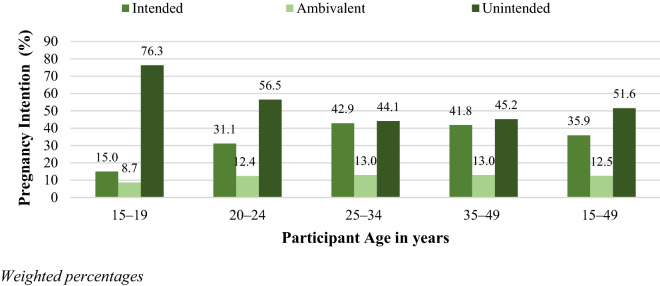
Pregnancy intention by age group in the 2019 Antenatal HIV Sentinel Survey, South Africa.

More than half (51.6% [95% CI: 50.9–52.4]) of the pregnancies in this study were unintended (Fig. 1; Supplemental Fig. 2). Just above one-third (35.9% [95% CI: 35.2%–36.7%]) of pregnancies were intended, and 12.5% (95% CI: 12.0%–13.0%) of participants were ambivalent about their pregnancies. The prevalence of unintended pregnancy was significantly higher among adolescent girls aged 15 − 19 years (76.3% [95% CI: 74.9%–77.6%]) and young women aged 20 − 24 years (56.5% [95% CI: 55.4%–57.6%]) compared to older women aged 35 − 49 years (45.2% [95% CI: 43.9%–46.6%]). Primigravid women had higher prevalence of unintended pregnancy (62.3% [95% CI: 61.3%–63.3%]) compared to multigravid women (46.9% [95% CI: 46.1%–47.7%]).

**Figure 2:**
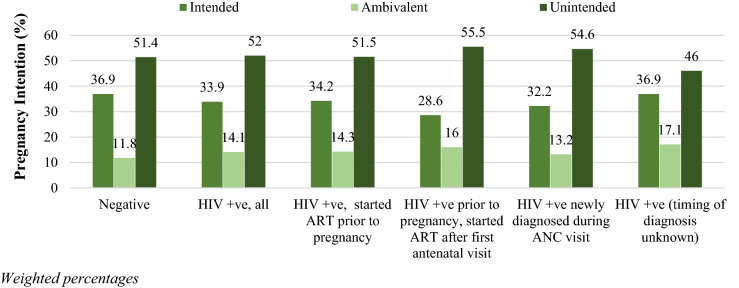
Pregnancy intention by HIV status in the 2019 Antenatal HIV Sentinel Survey, South Africa.

The overall prevalence of unintended pregnancy among HIV-positive women was 52.0% (95% CI: 51.0–53.0%) compared to 51.4% (95% CI: 50.6%–52.2%) among HIV-negative women (Fig. 2). Among HIV-positive women, the prevalence of unintended pregnancy among participants who knew their HIV-positive status before pregnancy but had not initiated ART until their first ANC visit was 55.5% [95% CI: 51.9–59.0%]. Participants who initiated ART before pregnancy had significantly lower unintended pregnancy rate (51.5% [95% CI: 50.3–52.7%]) compared to participants newly diagnosed with HIV during the ANC visit (54.6% [95% CI: 52.9–56.2%]).

Early (≤ 12 weeks) ANC attendance was significantly lower among participants whose pregnancy was unintended (32.9% [95% CI: 32.1–33.6%]) than among participants whose pregnancy was intended (38.9% [95% CI: 37.9–39.8%]) or who were ambivalent about their pregnancy (37.3% [95% CI: 36.0–38.6%]).

The prevalence of syphilis was not statistically different between participants with intended pregnancy (2.3% [95% CI: 2.1–2.6%]) and participants with unintended pregnancy (2.7% [95% CI: 2.4–3.0%]) or who were ambivalent about their pregnancies (2.7% [95% CI: 2.3–3.3%]).

On multivariable analysis (after adjusting for educational status), the odds of unintended pregnancy and being ambivalent about pregnancy was significantly higher among women who knew their HIV-positive status before pregnancy but had not initiated ART until after first ANC visit (unintended pregnancy: aOR, 1.5 [95% CI: 1.2–1.8]; ambivalent about pregnancy: aOR, 1.6 [95% CI: 1.2–2.1]), women who had initiated ART before pregnancy (unintended pregnancy: aOR, 1.3 [95% CI: 1.2–1.3]; ambivalent about pregnancy: aOR, 1.4 [95% CI: 1.3–1.5]) and women with a new HIV diagnosis during pregnancy (unintended pregnancy: aOR, 1.2 [95% CI: 1.1–1.3]; ambivalent about pregnancy: aOR, 1.2 [95% CI: 1.1–1.4]) compared to HIV-negative women (Table 3). Regarding HIV-positive women with unknown timing of HIV diagnosis, although based on the point estimate on Fig. 2 the prevalence of unintended pregnancy was lower in this group (compared to all other HIV/ART groups), once adjusted for confounding variables in a multivariable analysis (Table 3 ), there was no statistically significant difference in the prevalence of unintended pregnancy between this group and the reference group.

**Table 3:** Demographic and clinical characteristics associated with unintended and ambivalent pregnancy in the 2019 Antenatal HIV Sentinel Survey, South Africa (Reference group: Intended pregnancy).

	Prevalence of unintended pregnancy	Unintended pregnancy odds ratio (95% CI)		Ambivalent odds ratio (95% CI)	
	% (95% CI)	Crude	Adjusted	Crude	Adjusted
**Age among primigravida**					
15–19	79.0 (77.7–80.3)	8.5 (7.0–10.2)	6.9(5.7–8.4)	1.7 (1.4–2.2)	1.6 (1.2–2.0)
20–24	59.2 (57.8–60.6)	2.9 (2.4–3.5)	2.7(2.2–3.3)	1.3 (1.0–1.6)	1.2 (0.9–1.5)
25–29	42.2 (40.2–44.2)	1.4 (1.1–1.6)	1.3 (1.1–1.6)	0.9 (0.7–1.2)	0.9 (0.7–1.2)
30– 49	34.7 (31.3–38.2)	ref	ref	ref	Ref
**Age among multigravida**					
15–19	59.0 (55.4–62.4)	2.1 (1.8–2.4)	1.5 (1.3–1.8)	1.6 (1.2–2.0)	1.3 (1.0–1.7)
20–24	54.1(52.7–55.5)	1.6 (1.5–1.7)	1.3 (1.2–1.4)	1.3 (1.1–1.4)	1.1 (1.0–1.3)
25–29	46.3 (45.2 –47.4)	1.1 (1.1–1.2)	1.0 (0.9–1.1)	1.1 (1.0–1.2)	1.1 (1.0–1.2)
30–49	43.9 (42.9–44.9)	ref	ref	ref	Ref
**Gravidity among 15–19**					
Primigravida	79.0 (77.7–80.3)	2.9 (2.4–3.5_	2.3 (1.9–2.7)	1.7 (0.7–1.2)	1.2 (0.9–1.6)
Multigravida	59.0 (55.4–62.4)	ref	ref	ref	ref
**Gravidity among 20–24**					
Primigravida	59.2 (57.8–60.6)	1.3 (1.2–1.4)	1.1 (1.0–1.2)	1.3 (1.4–2.2)	1.0 (0.9–1.2)
Multigravida	54.1 (52.7–55.5)	ref	ref	ref	ref
**Gravidity among 25–29**					
Primigravida	42.2 (40.2–44.2)	0.9 (0.8–0.9)	0.7 (0.6–0.8)	0.9 (0.7–1.2)	0.9 (0.7–1.0)
Multigravida	46.3 (45.2–47.4)	ref	ref	ref	ref
**Gravidity among 30–49**					
Primigravida	34.6 (31.3–38.2)	0.7 (0.6–0.8)	0.5 (0.4–0.6)	1.2 (1.0–1.6)	1.0 (0.8–1.3)
Multigravida	43.9 (42.9–44.9)	ref	ref	ref	ref
**HIV status**					
Positive prior to pregnancy and in care (started ART prior to pregnancy)	51.5 (50.3–52.7)	1.4 (1.2–1.6)	1.3 (1.2–1.3)	1.8 (1.4–2.2)	1.4 (1.3–1.5)
Positive prior to pregnancy not in care (started ART during pregnancy)	55.5 (52.9–56.2)	1.2 (1.1–1.3)	1.5 (1.2–1.8)	1.3 (1.2–1.7)	1.6 (1.2–2.1)
Newly diagnosed with HIV during ANC visit	54.6 (52.9–56.2)	1.1(1.0–1.1)	1.2 (1.1–1.3)	1.1(1.2–1.4)	1.2 (1.1–1.4)
Positive (timing of diagnosis unknown)	46.0 (42.9–49.2)	0.9 (0.8–1.0)	1.0 (0.9 –1.2)	1.5 (1.2–1.7)	1.5 (1.2 –1.8)
Negative	51.4 (50.6–52.2)	ref	ref	ref	ref
**Relationship with the father of the child**					
Married	27.0 (26.0–28.1)	ref	ref	ref	ref
Co–habiting	41.4 (40.3–42.5)	2.1 (1.9–2.2)	1.9(1.8–2.1)	1.6 (1.4–1.7)	1.5 (1.4–1.7)
In a relationship, living apart	64.3 (63.4–65.2)	6.4 (6.0–6.8)	5.2(4.9–5.6)	3.1 (2.9–3.4)	2.9(2.6–3.1)
Single	76.0 (73.9 –78.0)	15.7(13.3–18.6)	12.9(10.7–15.4)	6.5 (5.2–8.1)	6.2(4.9–7.9)
**Gestational age at first visit**					
First trimester	47.8 (46.9–48.8)	ref	ref	ref	ref
Second trimester	53.1 (52.2–53.9)	1.3 (1.2–1.3)	1.2(1.2–1.3)	1.1 (0.9–1.1)	1.0(1.0–1.1)
Third trimester	62.7 (60.6–64.8)	2.0 (1.8–2.2)	2.1(1.8–2.3)	1.2 (1.1–1.5)	1.1 (0.9–1.5)

P value from Wald test for interaction term gravidity and age =  < 0.01; Weighted analysis. Missing data excluded. N = 30,194 observations (86.4% of data) included in multivariable analysis. The above model was adjusted for the woman’s education level.

Gravidity significantly affected the association between age and pregnancy intention (P value < 0.01). Women aged 15–24 years had higher odds of unintended pregnancy than women aged 30–49 years with this odds being excessively higher among primigravid women aged 15–19 years (aOR, 6.9 [95% CI: 5.7–8.4]), and 20–24 years (aOR, 2.7 [95% CI: 2.2–3.3]), compared to multigravid women aged 15–19 years (aOR, 1.5 [95% CI: 1.3–1.8]) , and 20–24 years (aOR, 1.3 [95% CI: 1.2–1.4]). Being primigravid was associated with a lower odds of unintended pregnancy compared to being multigravid for women aged 25–29 years (aOR, 0.7 [95% CI: 0.6–0.8]) or 30–49 years (aOR, 0.5 [95% CI: 0.4–0.6]).

Compared to married women, women in the following groups had significantly higher odds of unintended pregnancy or to be ambivalent about their pregnancy, respectively: single (aOR, 12.9 [95% CI: 10.7–15.4]; aOR, 6.2 [95% CI: 4.9–7.9]), in a non-cohabiting relationship (aOR, 5.2 [95% CI: 4.9–5.6]; aOR, 2.9 [95% CI: 2.6–3.1]), or in a cohabiting relationship (aOR, 1.9 [95% CI: 1.8–2.1]; aOR, 1.5 [95% CI: 1.4–1.7]).

Unintended pregnancy was significantly associated with late initiation of ANC; women who initiated their first ANC visit in the third trimester had two times higher odds of unintended pregnancy than women who initiated ANC in the first trimester (aOR, 2.1 [95% CI: 1.8–2.3]).

About 15% (4752/34,946) of participants were excluded from the multivariable model due to missing data for the individual variables included in the model. There was no statistically significant difference in demographic characteristics and HIV status between participants included and participants excluded from the multivariable model.

## Discussion

We found that one-half of pregnancies among women of reproductive age (15–49 years) and over three-fourths of pregnancies among adolescent girls and single women in South Africa are unintended. The risk of unintended pregnancy was higher among women who knew their HIV-positive status before pregnancy but had not initiated ART until after first ANC visit, women who initiated ART before pregnancy and women with a new HIV diagnosis during pregnancy, compared to HIV-negative women. Younger women (15–24 years) and unmarried women had higher risk of unintended pregnancy than older women and married women. Unintended pregnancy was associated with late initiation of ANC.

Although we found a higher odds of unintended pregnancy among HIV-positive women who initiated ART during or before pregnancy than HIV-negative women, this difference was modest. Our finding was inconsistent with a prior study conducted in Cape Town, which showed unintended pregnancy rates were 50% among HIV-positive women and 33% among HIV-negative women^11^. The main difference between the two studies was the lower prevalence of unintended pregnancy among HIV-negative women in the Cape Town study compared to our study, while the prevalence of unintended pregnancy among HIV-positive women was similar between the two studies. The later study (the Cape Town study) was conducted in 2015 in a province that has one of the lowest HIV burden and the highest uptake of contraceptive nationally^31^. Our study provides a more up-to-date and generalizable data nationally than the Cape Town study. Another study that has done a meta-analysis of studies conducted between 2015 and 2016 in SSA countries showed HIV-positive women had a higher prevalence of unintended pregnancy than HIV-negative women^32^. However, given that different instruments (including non-validated instruments) were used for the measurement of unintended pregnancy in the studies included in the above meta analysis study, it will be difficult to compare the findings of those studies with our study.

Although the difference in the prevalence of unintended pregnancy between HIV-positive and HIV-negative women was modest in our study, unintended pregnancy could have additional health risk for HIV-positive women and their babies. Because HIV-positive women receiving ART visit health facilities for ART refill more frequently (every 3 months at the time of this study) than HIV-negative women, discussing family planning and available contraception options during ART refill visits could help decrease unintended pregnancy rates among HIV-positive women^33–36^.

The high prevalence of unintended pregnancy among women with a new HIV-positive diagnosis suggests that addressing barriers that lead to unprotected sexual intercourse can prevent both unintended pregnancy and HIV infection. This finding highlights the need to integrate messages on dual protection into community-based HIV campaigns to address clients’ needs holistically to prevent both HIV/STIs and unintended pregnancy^11^. Targeting both men and women with awareness campaigns could be effective because men may be involved in reproductive health decision making^37,38^. The high prevalence of unintended pregnancy among women who knew their HIV-positive status before pregnancy but who had not initiated ART highlights the importance of linkage-to-care after an HIV diagnosis to provide both HIV treatment and reproductive health services^11^.

Our finding that half of the pregnancies were unintended among our participants was consistent with findings from other studies^31,39^. Data from the South African Demographic and Health Survey (SADHS) reported 54% of all births between 2012 and 2016 were unintended^31^. Comparison of our findings with the SADHS also revealed, positive correlation, in most provinces, between the prevalence of unintended pregnancy (in our study) and the prevalence of unmet family planning need (in SADHS)^40^. Comparison of our findings with a study that assessed unintended pregnancies in sub-Saharan Africa showed that South Africa had the second highest prevalence of unintended pregnancy in the sub-Saharan Africa region^41^. Given the higher prevalence of late initiation of ANC among HIV-positive women with unintended pregnancy, this may also present a barrier to efforts to eliminate mother-to-child HIV transmission^42,43^. Addressing key challenges and gaps that contribute to unintended pregnancy could decrease the current high rate of unintended pregnancy.

Low uptake of contraception has been reported as the cause of up to 75% of unintended pregnancies in South Africa^39,44^. Fear of side effects leading to discontinuation of contraception and fear of infertility are two frequently cited reasons for not using contraception in the literature; however, these reasons could indicate inadequate knowledge about the various safe and reversible contraception options available^39,40,45–48^.

In other African countries, community-based services are increasingly being used to address misconceptions and concerns about contraception within the community, along with provision of commonly used contraceptive methods at community level, which have been shown to improve uptake of contraception significantly^49–51^. In South Africa, free contraceptive services are available in family planning clinics at all health service levels^52^. Injections and pills are commonly used contraceptive methods in South Africa. Although, the guideline recommends that a wide range of contraceptive options should be available at primary health care facilities, often due to stock outs and limited (health care provider and user) knowledge about the different contraceptive methods available, the use of hormonal implants and intrauterine devices is low in South Africa^52^. Extending contraceptive services outside of family planning clinics (e.g., in schools, abortion clinics, and mobile outreach services) in South Africa could help increase uptake of contraception^53^. It is also essential to address supply-chain disruptions and the knowledge gap among health care providers and users^54^.

Consistent with other studies, in our study, women aged 15–24 years and single women had disproportionately high unintended pregnancy rates. The literature shows that the high unintended pregnancy among adolescent girls could be attributed to underlying and interconnected factors, such as poverty and poor mental health, that increase the vulnerability of young women, as well as low knowledge of contraceptive methods, fear of using contraception (due to parental disapproval), male partner influence, and stigma associated with accessing reproductive health services^40,48,55–57^. Several interventions are currently being implemented in pilot sites in South Africa to increase contraception use among adolescent girls and young women: the She Conquers and DREAMS initiatives, adolescent and youth friendly services, and school health nurse initiatives^58–60^. Assessing the impact of these interventions could help scale-up successful interventions nationally. The lower risk of unintended pregnancy among multigravid adolescent and young women compared to primigravid women may be due to the exposure of multigravid women to counselling and contraceptive services during previous pregnancies.

Our study has some strengths and limitations. The percentage distribution of pregnant women by age group in this study was consistent with the age distribution reported for pregnant women in the SADHS 1998 and 2016 surveys (where the highest percentage of pregnancies were reported in the age groups 20–24 and 25–29 years) confirming that our sample is representative of the South African pregnant women population^31^. Therefore our study provides generalizable estimates on the prevalence of unintended pregnancy among HIV-positive and HIV-negative women at the national and sub-population level. Our findings do not apply to women who use ANC services in the private health sector because our study was limited to public clinics. Excluding private facilities may result in overestimation of the prevalence of unintended pregnancy because women who attend private facilities are likely to be middle-income or high-income and may have lower prevalence of unintended pregnancy^41^. In contrast, our study excluded women who had terminated their pregnancies who may have higher prevalence of unintended pregnancy, which may have resulted in underestimation of unintended pregnancy. The LMUP questions used in this survey are part of a validated measure of pregnancy intention; however, because our study used only two LMUP questions, our estimates may not be directly comparable with findings from other studies that used all six LMUP questions. In this study, the estimated unintended pregnancy prevalence per the women’s response for the partner question (LMUP 5) was to a large extent similar to the women’s intention (LMUP 3). Given that both questions were answered by the women, the response could be biased towards the women’s intention. In the literature, studies show inferences about partner intention as measured by the report of the pregnant women may not reliably represent the partner’s intention^61,62^. Therefore, our estimate on partner involvement should be interpreted with caution. Given that health workers often disapprove adolescent pregnancy^63^, adolescent girls may under report their true pregnancy intention in fear of disapproval by the nurses collecting this data—in this case, the true prevalence of unintended pregnancy among adolescent girls could be higher than the reported prevalence in this study. Because our study collected limited data on demographic and behavioral factors that may influence unintended pregnancy, the identified factors associated with unintended pregnancy may not be a comprehensive list.

In conclusion, our study showed high unintended pregnancy rates across population groups, with the highest prevalence among young women aged 15–24 years, unmarried women, women who knew their HIV-positive status before pregnancy but had not initiated ART until after their first ANC visit, women in care before pregnancy and newly diagnosed women. Implementing adolescent and youth friendly services, school health nurse initiatives, and consistent and intensive public education campaigns to raise awareness; strengthening integration of HIV and contraceptive services (including regular assessment of HIV clients’ pregnancy intention and providing contraceptive services at HIV testing/treatment sites) and linkage-to-care after HIV diagnosis; and increasing access to a wide range of contraception options could help address the unmet need for contraception and the high unintended pregnancy rate in South Africa.

## Supplementary Information

Supplementary Information.

## Data Availability

Data cannot be shared publicly because the data is owned by a third party. Data are available from the National Health Laboratory Services Academic Affairs and Research Unit (contact via academic.research@nhls.ac.za.) for researchers who meet the criteria for access to confidential data.
