# Supplementary material for: The prevalence of unintended pregnancy and its association with HIV status among pregnant women in South Africa, a national antenatal survey, 2019
Source: Sci Rep. 2021 Dec 9;11:23740. doi: 10.1038/s41598-021-03096-z (PMC8660789; doi:10.1038/s41598-021-03096-z)
Supplement: Supplementary file 1 — Supplementary Information. [file 41598_2021_3096_MOESM1_ESM.docx]

**Supplementary Table 1**

**The Scoring Method**

**Table 1:** London measure of unplanned pregnancy (LMUP) scoring applied in the national antenatal HIV sentinel survey planning of pregnancy questions.

| **Response to the London measure of unplanned pregnancy (LMUP) questions** | **Score** |
| --- | --- |
| Just before I (mother) became pregnant:  I intended to become pregnant  My intention kept changing  I did not intend to be pregnant | 2  1  0 |
| Before I became pregnant:  The father of the child and I (mother) had agreed that we would like me to be pregnant    The father of the child and I (mother) had discussed having children together but hadn’t agreed for me to be pregnant  We never discussed having child(ren) together | 2  1  0 |

The pregnancy intention questions were analyzed using the following strategy: the two LMUP questions included to assess the degree of planning of pregnancy were each given a score of 0, 1, or 2 (as illustrated in Table 1), and the scores from the two questions were added together to give an ordinal scale ranging from 0 to 4. To estimate the prevalence of unintended pregnancy, scores 0 and 1 (either response represented unintended pregnancy) were categorized as “unintended pregnancy,” scores 2 and 3 (which represented planning by one response or undecided intention) were categorized as “ambivalent (undecided),” and score 4 (which represented intention to be pregnant by both questions) was categorized as “intended pregnancy.” The results from this analysis is presented in supplementary figure 1

**Supplementary Figure 1:** Pregnancy intention by age group in the 2019 antenatal HIV sentinel survey, South Africa using the scoring method

**Supplementary Figure 2**

EC=Eastern Cape, FS=Free State, GP= Gauteng Province, KZN=KwaZulu Natal, LP = Limpopo Province, MP= Mpumalanga Province, NW= North West, NC= Northern Cape, WC= Western Cape

**Supplementary Figure 2:** Unintended pregnancy by province and HIV status in South Africa, the 2019 Antenatal HIV Sentinel Survey
